# Determination of breeding criteria for gait proficiency in leisure riding and racing dromedary camels: a stepwise multivariate analysis of factors predicting overall biomechanical performance

**DOI:** 10.3389/fvets.2023.1297430

**Published:** 2024-01-16

**Authors:** Carlos Iglesias Pastrana, Francisco Javier Navas González, Elena Ciani, Carmen Marín Navas, Juan Vicente Delgado Bermejo

**Affiliations:** ^1^Department of Genetics, Faculty of Veterinary Sciences, University of Cordoba, Cordoba, Spain; ^2^Department of Biosciences, Biotechnologies and Environment, University of Bari ‘Aldo Moro’, Bari, Italy

**Keywords:** gait performance, quantitative genetics, curve estimation regression models, discriminant analysis, breeding criteria, dromedary camel

## Abstract

To date, the biomechanical dynamics in camelids have not been addressed, although it might be a factor that can affect selection and breeding in this species. Therefore, the aim of this article is to conduct curve fitting and discriminant canonical analysis to identify the mathematical function that best captures the dynamics of camel locomotion and to study the impact of kinematic, morphometric, physiological, and phaneroptic variables on gait performance in leisure riding and racing activities in dromedaries, respectively. The cubic function emerged as the most suitable mathematical model to represent the locomotive behavior of camels. Various factors were found to play a pivotal role in the athletic performance of leisure riding and racing dromedary camels. Concretely, angular measurements at the distal fore and rear extremity areas, pelvis inclination, relative volume of the hump, impact forces of the front limbs, post-neutering effects, and the kinematic behavior of the scapula, shoulder, carpus, hip, and foot are the factors that greatly impact gait performance in leisure riding and racing camels. The biomechanical performance at these specific body regions has a profound impact on weight absorption and minimization of mechanic impact during camel locomotion, static/dynamic balance, force distribution, energy of propulsion, movement direction and amplitude, and storage of elastic strain in leisure riding and racing dromedaries. In contrast, other animal- and environment-dependent factors do not exert significant influence on camel gait performance, which can be attributed to species-specific, inherited adaptations developed in response to desert conditions, including the pacing gait, broad foot pads, and energy-efficient movements. The outcomes of our functional data analysis can provide valuable insights for making informed breeding decisions aimed at enhancing animal functional performance in camel riding and racing activities. Furthermore, these findings can open avenues for exploring alternative applications, such as camel-assisted therapy.

## 1 Introduction

Although dromedary camels (*Camelus dromedarius*) have been present in the Iberian peninsula since the Roman period (1), the Canarian archipelago is the only Spanish national territory where history, culture, and ecology of these animals have been strongly rooted for more than 600 years (2). The oldest records of the presence of dromedaries in the archipelago coincide with the historical chronicles relating to the process of European colonization of the eastern islands (3). Due to its anatomical-physiological characteristics and its outstanding performance in fatigued draft works in the arid territories of the islands (4, 5), the camel has managed to perpetuate itself in them until the present.

For centuries, the possession of a camel among local farmers was considered a symbol of status and social prosperity as well as a reinforcement of the family livelihood. Unfortunately, the mechanization of agriculture from the last third of the 20th century led to the progressive substitution of camels in rural labor and the migration of entrepreneurs to the tourism sector. Thus, the initiation of the activity of the National Paradors in the late 1950s and the emergence of tourism as an economic activity in the archipelago contributed to the regression of the camel census in rural areas and their functional reorientation. That is, the main productive niche of the camel in the Canary Islands has since become tourist leisure, in which both animal behavior and physical conformation/performance have a notable impact (6).

Considering the census of active breeders, the risk status assigned to the breed was endangered, a condition still in force today (Order AAA/251/2012; Spanish Ministry of Agriculture, Food and Environment). The lack of knowledge about the productive potentials and the underutilization of local animal genetic resources at risk of extinction places them in a scenario of special vulnerability. Although the potential of this camel breed for milk production purposes is preliminary evaluated, camelback leisure riding continues to be the most profitable activity for this camel breed, which has also expanded its geographical distribution to other European countries during the last three decades in an attempt to mitigate the risk of extinction under the potential occurrence of local stochastic phenomena. In this context, the investigation and promotion of the current and future potential of these resources to ensure the sustenance of human beings and the balance of the local environment will help promote existing productive niches and define new ones.

The observable traits expressed by an organism are mainly governed by the inter-locus interaction (epistasis) of alleles for multiple single genes (7). In livestock scenarios, the magnitude and impact of such complex genetic interactions are driven to a great extent both by historical and current uses of animal species and the design and purposes of breeding programs. Indeed, epistatic genes are agreed to be a major feature in the genetic architecture of evolutionarily transcendent phenotypic variation (8). For the particular case of the relationships between physical attributes and gait performance in domestic animal species involved in moderately energy-demanding sports activities, scarce empirical information does exist. As such, selective breeding schemes may fail to reach genetic improvement and interfere with the adaptive natural history of the animals (9).

In the particular case of the Canarian camel, the present study pursues the biomechanical characterization of the breed by means of 2D video captures. Previous related research has performed basic analyses of the biomechanics of camel gaits (10–12) and the elastic extension of tendons (13), identified some environmental factors (e.g., sex and age) affecting the racing performance (14), and studied the morphology of some parts of the distal skeleton (10, 15–17) and the pedal anatomy (18). However, no reference literature reports the exercise- and animal morphometrics and phaneroptic-related factors that are potentially responsible for oscillations in the functional performance of camel locomotion to any degree of accuracy. Thus, the current research primarily aims to perform individual curve estimations through regression analysis to identify the model that best fits camel biomechanics when performing leisure riding or racing activities, for which camel pace is the most prevalent gait (19). Afterward, such curve fitting or functional data analysis approach will solve the need for stakeholders and breeders to identify a model to describe the dynamics of camel locomotion and detect the location and magnitude of differences in gait performance within the evaluated function (20). Following the theoretical body by Saastamoinen and Barrey (21) on the genetics of horse locomotion, this study also hypothesizes that the locomotor performance in dromedary camels is the overall result of the combination of conformational, physiological, and behavioral heritable traits. Indeed, the selection supported by these characters could overcome the generally low heritability of performance traits (22). Hence, after identifying the mathematical model that best describes camel locomotion, this study pursues the identification of the kinematics, animal morphometrics, physiological, and phaneroptic-related variables that significantly impact the gait proficiency of dromedaries in leisure riding and racing activities. The results obtained will aid in refining animal selection strategies for athletic performance in leisure riding and racing activities, as well as in the exploration of different alternatives in which to venture (i.e., assisted therapy) and with which to reinforce the tasks of functional valorization for its sustainable conservation. The methodology proposed is also expected to be translatable in a comparative manner to other camel populations involved in athletic and draft activities, for which these activities constitute the most profitable niches.

## 2 Materials and methods

### 2.1 Animal sample and data purge

A total of 130 Canarian dromedaries (72 male and 58 female camels; aged between 18 months and 35 years) were included in this study. These camels were located in three representative breeding locations within Spain: Huelva (Doñana National Park, coordinates 36.972330, −6.427498), Almería (coordinates 36.902180, −2.429520), and Fuerteventura (coordinates 28.186777, −14.158361). Gait evaluation was performed on all the animals, but data collection and extraction were only feasible in those animals that, first, were able to develop any of the three specific gaits performed by dromedaries as described in the literature, namely, walk, pace, and gallop; and second, from which two complete stride cycles could be collected and extracted. Animals that did not fulfill the aforementioned requirements were discarded from the database for further analysis. All animals were deemed healthy after clinical examination by a trained practitioner. No signs of pain or thoracolumbar vertebral alterations were observed.

As a result of this purging process, the number of animals producing data to be considered in statistical evaluations is as follows: 82 animals for walk, 97 for pace, and 10 for gallop. There were some animals (*n* = 60) that could be evaluated for more than one type of gait. The reduced number of animals that engaged in galloping patterns may be ascribed to the fact that, as described in the literature (18, 23, 24), these animals are inner energetic savers and hence rarely engage in faster patterns of movements except for very concrete situations in which their life may be compromised or threatened, or if trained to do it so as it happens in trained, racing dromedaries. In fact, according to Tharwat et al. (19), although at the beginning of the race most camels gallop, they frequently switch between pacing and galloping throughout the competition. It is intriguing to note that camels can maintain a pace that is nearly as swift as their gallop.

### 2.2 Biometric variables and force mathematical determination

The development of gaits has been reported to considerably depend not only on the biometry of dromedaries but also on the relative forces that the animals are able to develop. For these reasons, the following variables were considered in the present study.

First, live weight was calculated using the formula by Boujenane (25). Afterward, pull force (maximum and minimum), load force (maximum and minimum), and maximum and minimum power were calculated following the methods described by Delgado et al. (26).

Smoothened movement parameters are adjusted to trigonometric functions based on specific anatomical regions in both the forelimbs and hindlimbs. Given the implication of angles shared between joints, eight angles were considered: angle 1 = cranial angle of the scapula-midway between acromion and head of humerus; angle 2 = midway between acromion and head of humerus-olecranon; angle 3 = olecranon-carpus; angle 4 = carpus-fetlock (metacarpophalangeal joint) on the forelimb; angle 5 = iliac crest-greater trochanter of the femur; angle 6 = greater trochanter of the femur-stifle (knee) joint; angle 7 = knee joint-tarsus; and angle 8 = tarsus-fetlock (metatarsophalangeal joint) on the hindlimb.

The calculation of hump volume (HV = 0.07 L × B × H), hump weight (HW = 0.45 H – 13.8), the ratio HW/HV (fat density), the proportion HV/BW, and the proportion HW/BW was performed using the methods described by Bengoumi et al. (27). Additional indexes reported to condition the quality of movement and kinetics were also calculated, including chest index, cephalic index, pelvic index, dactilothoracic index, weight in cannon index, conformation index, chest height index, and compactness index, upon the methods described by Susana Lopes et al. (28).

The units of measurement for biokinematics variables were set using Dunbar et al. (29) as a reference.

### 2.3 Experimental setup and video recording

The image collection occurred on a level and solid open ground. Specific lighting conditions were selected to ensure that the animal was not situated in shadowed areas or under lighting that could distort the image capture. Additionally, the color of the animal was taken into account to prevent any potential distortion or misalignment caused by the background color.

To maintain consistency, the camera was positioned at a standardized height of 1 m on a camera stand. This setup was situated 4 m away from the center of balance of the camel. This configuration allowed for capturing the entire animal within the frame during the evaluation. To ensure proper alignment, we followed the guidelines presented by Iglesias et al. (30), which involved marking standard lines on the ground before taking photographs to confirm the animal’s correct positioning.

The image capture process utilized a digital camera (Sony DSC-RX100 SENSOR CMOS Exmor 1.0, 20.1 MP, F1.8–4.9, Zoom 20–100, Optical Zoom 3.6×, 3″ LCD Screen with Image Stabilizer; Sony Electronics, San Diego, CA, United States) in its standard mode. Cameras are strategically positioned to capture the observations without interfering with the animal’s movement along the evaluation track. Dromedaries were recorded from two different views (side view and front view). The images were saved using the Joint Photographic Experts Group (JPEG) compression format.

**Figure 1:**
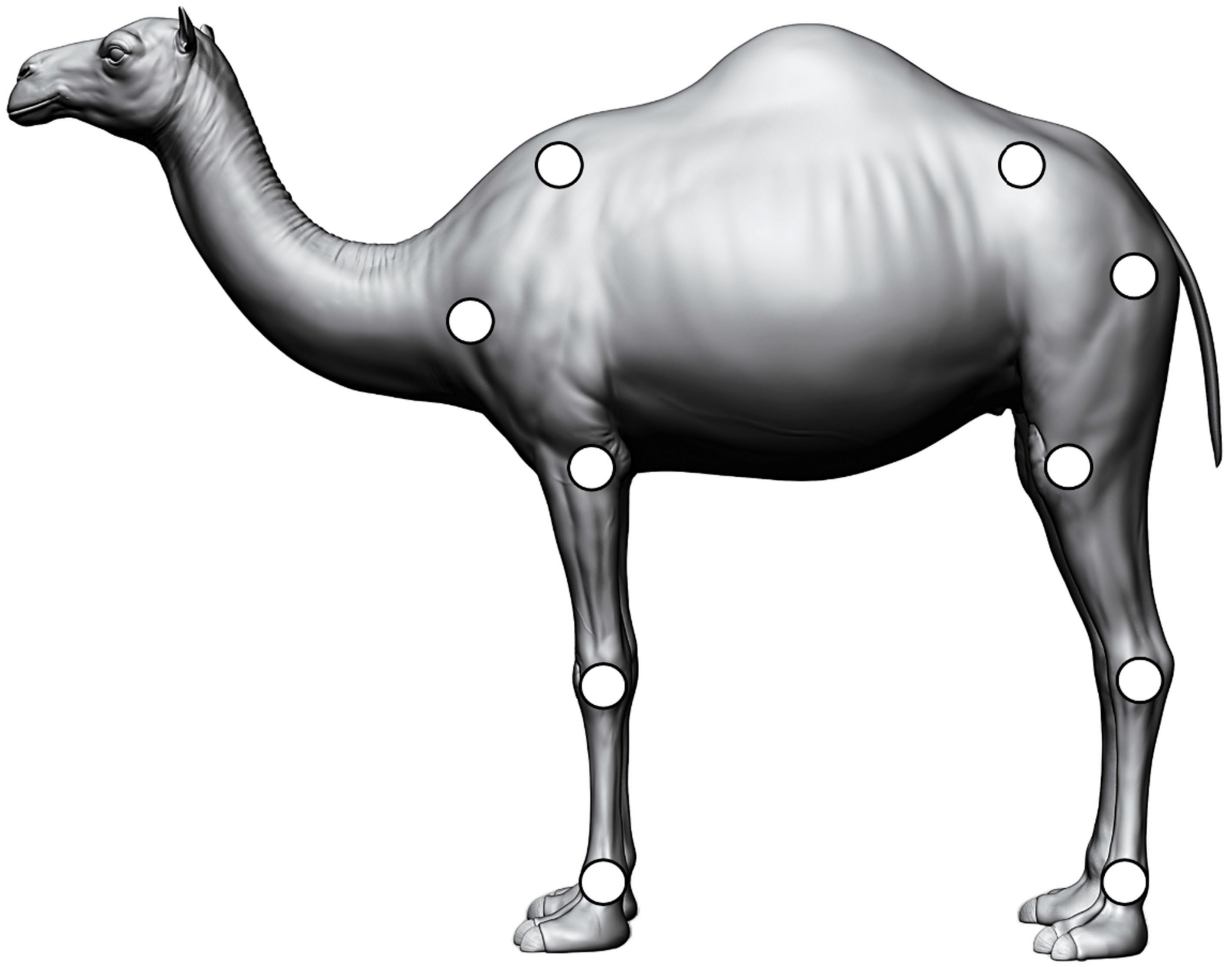
Graphical illustration of the anatomical disposition of masking tape squares (5 cm × 5 cm) at the dromedaries’ bodies to serve as joint tracers reference points for gait analysis.

Before recording, body length and height were measured, and masking tape squares (5 cm × 5 cm) were affixed to the dromedaries’ bodies to serve as joint tracer reference points for data analysis. These markers were placed on the following left-side anatomical landmarks of the dromedaries: cranial angle of the scapula, midway between acromion and head of the humerus (shoulder joint), olecranon (elbow joint), carpus and fetlock (metacarpophalangeal joint) on the forelimb, the iliac crest, greater trochanter of the femur (hip), stifle (knee) joint, point of the hock (tarsus), and fetlock (metatarsophalangeal joint) on the hindlimb, as shown in Figure 1. A trained operator visualized all video sequences and placed video tracers from video analysis software Kinovea software (Kinovea version 0.9.5; Kinovea Org., France). The same operator performed the potential replacement of joint tracers along the video sequences when a disagreement between the real course and the course of the point of the software occurred.

A wooden pole (100 cm) was positioned parallel to the gait plane to provide a horizontal reference and calibration. The camera, mounted on a tripod, was adjusted to minimize parallax and ensure level alignment. Video footage was collected while the dromedaries were engaged in their regular exercise routines, encompassing walking, pacing, and galloping.

### 2.4 Qualitative and quantitative stride-based gait analyses

**Figure 2:**
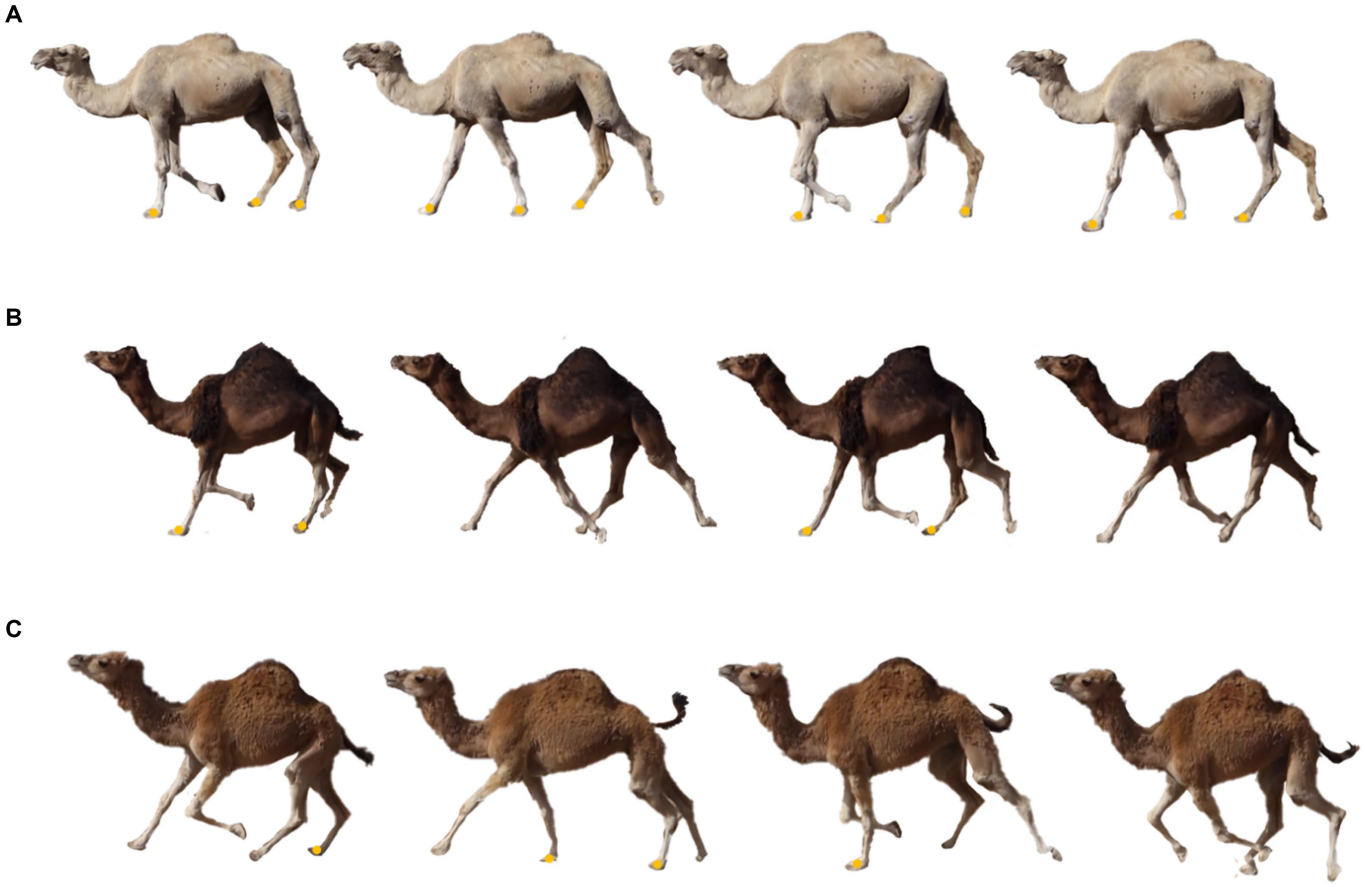
Graphical representation of the gait cycle during the walk **(A)**, pace **(B)**, and gallop **(C)** in dromedary camels. Yellow dots indicate which limbs are on the ground at each phase of the dromedary gait cycle.

The recording duration corresponds to the time taken by the animal to complete the circuit for each of the three gaits examined (walking, pacing, and galloping), as shown in Figure 2. Two complete stride cycles were considered for each animal. Stride-based gait analysis generally considers the contact of the trailing hindfoot as the starting point of the stride cycle (31). In the relative limb phase, the fraction of a stride in which the left forefoot, right hindfoot, and right forefoot touchdown was calculated following the left hindfoot touchdown. It was calculated by counting the frames of the filmed motion sequences based on the method by Hildebrand (32).

The individual locomotion process is assessed using a qualitative linear scale (1 to 5 points) described by Navas González et al. (33). Gaits characterized by a lack of uniformity, which may be indicative of an absence of balance, cadence, or harmony, and poor limb development were attributed a score of 1. In contrast, animals receiving a score of 5 were observed to exhibit a harmonious, rhythmic, and seamlessly coordinated gait, with their bodily movements demonstrating a high degree of synchronicity.

Kinovea software (Kinovea version 0.9.5; Kinovea Org., France) was used to analyze video sequences and kinematic data extraction. With Kinovea, images are calibrated using coordinate scales from the pictures, and the collected data undergo leveling through three smoothing methods. Eleven kinematic variables were considered to characterize each of the gaits performed by the camels as a default. Biomechanics parameters were as follows: acceleration, horizontal acceleration/position/velocity, total distance, total horizontal/vertical displacement, velocity, and vertical acceleration/position/velocity. An experienced researcher digitized anatomical landmarks to define body segments, including the lower lip, ear, withers (third thoracic vertebra), and tail base (dock). Segments’ axes, such as the head, neck, and trunk, were established using these landmarks. Quantitative analysis was performed on trials where the head, neck, and trunk remained aligned in the sagittal plane. Various linear and angular variables related to the head movement, including displacement and velocity, were calculated using specialized software tools.

### 2.5 Definition of cubic regression equations and coefficients

Individual curve estimation regression analysis to identify the model that best describes camel biomechanics parameters (acceleration, horizontal acceleration/position/velocity, total distance, total horizontal/vertical displacement, velocity, and vertical acceleration/position/velocity) was performed using the *Curve Estimation* routine of the *Regression* package of SPSS Statistics for Windows, Version 25.0, IBM Corp. (2017). The *Curve Estimation* routine in SPSS software produces curve estimation regression statistics and related plots for 11 different curve estimation regression models, such as linear, quadratic, compound, growth, logarithmic, cubic, S, exponential, inverse, power, and logistic,. A generalized minimum adjusted *R*^2^ value of over 0.7 was required for the model to be determined valid to capture data variability. The mathematical model that best fitted (comparatively superior average values of individual *R*^2^; >0.7) the dynamics of locomotion of study animals was the cubic model. In these regards, parameters β0, β1, β2, and β3 of the curve for the variables of acceleration, horizontal acceleration/position/velocity, total distance, total horizontal/vertical displacement, velocity, and vertical acceleration/position/velocity, respectively, for each of the limbs were considered as independent variables in discriminant analysis.

In a cubic regression model, the equation takes the form:


$$y=\beta 0+\beta 1x+\beta 2x^{2}+\beta 3x^{3}+\epsilon$$

Where:

*β*0: This is the intercept term and represents the predicted value of *y* when *x* is 0. However, the interpretation of this term might not be as straightforward in some cases, depending on the range of your data and the context of your problem.

*β*1: This coefficient represents the change in the predicted *y* for a one-unit change in the linear term *x*. It captures the linear relationship between *x* and *y*, just like in a simple linear regression model.

*β*2: This coefficient represents the effect of the squared *x* on the predicted *y*. It indicates whether there is a curvature in the relationship between *x* and *y*. A positive *β*2 suggests an upward curvature, while a negative *β*2 suggests a downward curvature.

*β*3: This coefficient represents the effect of the cubed *x* on the predicted *y*. It introduces another level of curvature to the relationship between *x* and *y*. A positive *β*3 suggests that the curvature continues to be upward, while a negative *β*3 suggests a downward curvature.

### 2.6 Statistical analysis

Following the methodology by González Ariza et al. (34), a discriminant canonical analysis was first used to develop a tool that enables the identification of the qualitative individual performance at locomotion (1 to 5 points; clustering criterion), determining whether linear combinations of biomechanics-related and biometrics variables, force and cubic regression coefficients, and categories for the variables “sex”, “coat color”, “coat particularities”, “eye color”, “neutering status”, “owner”, and “training regime” (actively or not actively involved in desensitization protocols) (explanatory variables) describe within- and between-population group clustering patterns. The discriminant canonical analysis is a statistical method that maximizes the discriminatory power of the variables, enhancing the accuracy of the results even with a relatively reduced number of observations (e.g., endangered livestock breeds). This technique has a notable ability to handle multicollinearity and identify the most influential variables that contribute to its accuracy, especially in situations where limited sample sizes may pose challenges for other statistical approaches.

The chi-squared automatic interaction detection (CHAID) decision tree method was used for classification, prediction, interpretation, and manipulation of the observation for the aforementioned independent or explanatory variables discretely categorized into the qualitative evaluation levels of gaits considered as the dependent variables of this analysis.

To assess the reliability of the CHAID decision tree, cross-validation is conducted to measure the predictive performance when applied to new data samples compared to the training sample. This assessment helps determine how well the model generalizes to unseen data. To validate the CHAID decision tree and evaluate whether the selected predictors effectively explain differences across the five qualitative evaluation levels of gaits, 10-fold cross-validation is utilized.

## 3 Results

### 3.1 Discriminant canonical analysis model reliability

After 325 rounds of multicollinearity analyses, the variables retained in the discriminant canonical analyses are those presented in Table 1.

**Table 1:** Summary of the value of tolerance and VIF after multicollinearity analysis of biomechanical performance traits in Canarian camel breed.

Statistic	Tolerance (1−*R*^2^)	VIF (1/Tolerance)
Angle 4	0.607	1.646
Proportion HV/BW	0.538	1.859
B3-Scapula-Horizontal Acceleration	0.517	1.934
Angle 7	0.516	1.939
Angle 5	0.511	1.958
Chest height index	0.469	2.131
Weight in the cannon index	0.447	2.238
B1-Iliac crest-Horizontal Acceleration	0.443	2.255
B2-Fore fetlock-Vertical Acceleration	0.432	2.315
B2-Scapula-Horizontal velocity	0.425	2.353
B1-Hind fetlock-Vertical velocity	0.424	2.361
B2-Carpus-Total Vertical displacement	0.393	2.547
B1-Knee-Vertical acceleration	0.391	2.557
B3-Shoulder-Acceleration	0.367	2.727
B2-Hip-Horizontal Velocity	0.355	2.820
B2-Elbow-Acceleration	0.354	2.824
Neutered-No	0.344	2.908
B2-Shoulder-Horizontal position	0.342	2.927
B2-Scapula-Horizontal position	0.337	2.964
B2-Iliac crest-Horizontal velocity	0.321	3.117
B2-Scapula-Total vertical displacement	0.317	3.154
B1-Hip-Horizontal position	0.316	3.166
B2-Shoulder-Vertical velocity	0.315	3.177
B2-Iliac crest-Horizontal position	0.308	3.243
B1-Knee-Total vertical displacement	0.295	3.388
B2-Shoulder-Horizontal velocity	0.292	3.429
B2-Fore fetlock-Acceleration	0.291	3.442
B3-Hip-Vertical velocity	0.286	3.497
Body ratio	0.285	3.514
B2-Hip-Total vertical displacement	0.275	3.641
B1-Hip-Horizontal Acceleration	0.273	3.666
B1-Carpus-Vertical velocity	0.260	3.844
B2-Iliac crest-Total vertical displacement	0.257	3.897
B2-Hip-Vertical acceleration	0.255	3.925
B3-Knee-Vertical velocity	0.254	3.941
B2-Hind fetlock-Vertical acceleration	0.250	3.995
B1-Elbow-Vertical acceleration	0.249	4.021
B1-Elbow-Vertical velocity	0.247	4.050
B3-Iliac crest-Vertical velocity	0.240	4.166
B2-Elbow-Horizontal velocity	0.238	4.208
B2-Knee-Acceleration	0.234	4.276
B1-Scapula-Vertical acceleration	0.231	4.321
B1-Hind fetlock-Total vertical displacement	0.217	4.606
B2-Tarsus-Horizontal velocity	0.212	4.713

Interpretation thumb rule: VIF ≥ 5 (highly correlated); 1 < VIF < 5 (moderately correlated); VIF > 1 (not correlated).

As unbalanced samples and a considerable number of variables were used, the approximation of Rao for Wilks’ Lambda test overcomes the robustness of other tests, such as Pillai’s trace test statistic in the case of heterogeneous variance. Wilks’ Lambda statistic was highly statistically significant when Gait qualitative scale levels (Wilks’ Lambda statistic: 0.001; F (Observed value): 4.202; F (Critical value): 3.258; df1: 736; df2: 7; *p* = 0.025) were considered clustering criteria. Hence, the validity of the discriminant canonical analysis is ensured.

**Figure 3:**
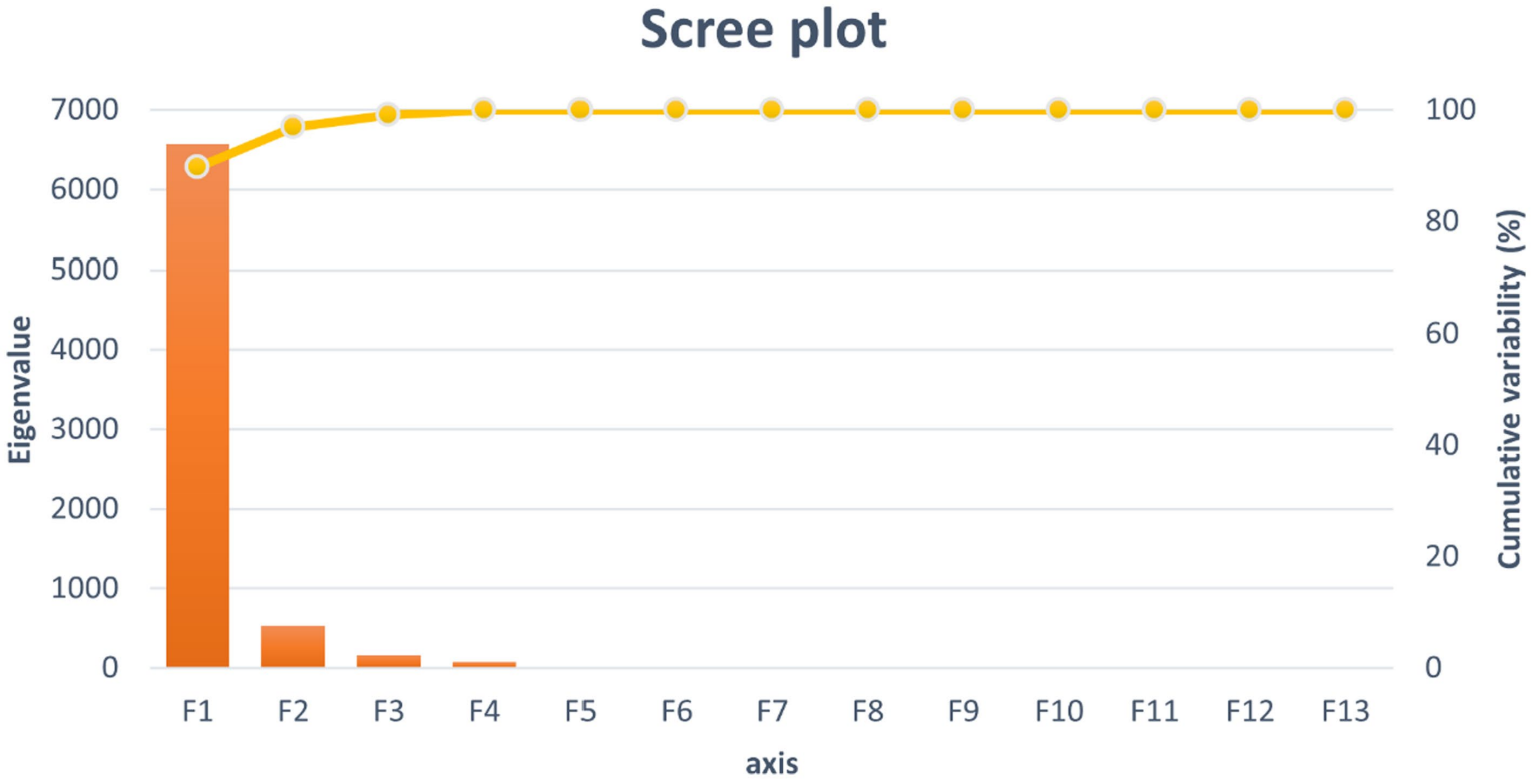
Canonical variable functions and their percentages of self-explained and cumulative variance.

Five of the thirteen functions revealed after the discriminant analysis were reported to be significant for their discriminant ability (Table 2). The discriminatory power of the F1 function was the highest in comparison to that reported for the rest of the functions (eigenvalue of 6568.067; Figure 3), significantly explaining 89.72% of the variance. This framework makes F1 to F4 significantly explain 100% of the variability.

**Table 2:** Canonical discriminant analysis efficiency parameters to determine the significance of each canonical discriminant function.

Test functions	Canonical correlations	Eigenvalue	Variance discrimination (%)	Bartlett’s statistic	*p*-value
1 through 13	1.000	6568.067	89.720	2281.745	0.001
2 through 13	0.999	525.554	7.179	1459.868	0.001
3 through 13	0.997	153.996	2.104	873.964	0.001
4 through 13	0.993	72.981	0.997	402.406	0.001
5 through 13	0.000	0.000	0.000	0.000	0.001
6 through 13	0.000	0.000	0.000	0.000	1.000
7 through 13	0.000	0.000	0.000	0.000	1.000
8 through 13	0.000	0.000	0.000	0.000	1.000
9 through 13	0.000	0.000	0.000	0.000	1.000
10 through 13	0.000	0.000	0.000	0.000	1.000
11 through 13	0.000	0.000	0.000	0.000	1.000
12 through 13	0.000	0.000	0.000	0.000	1.000
13	0.000	0.000	0.000	0.000	1.000

### 3.2 Canonical coefficients and loading interpretation

The different variables studied in this research were ranked according to their discriminating ability. A test of equality of group means of the independent variables involved in the present discriminant canonical analysis was used, as shown in Table 3. Standardized discriminant coefficients measure the relative weight of each independent variable across the significant discriminant functions (Supplementary Table S1).

**Table 3:** Results for the tests of equality of group means to test for difference in the means across sample groups after the removal of redundant variables.

Variable	Wilk’s Lambda	*F*	*p*-value	Rank
B1-Elbow-Vertical velocity	0.769	13.822	<0.0001	1
B2-Iliac crest-Horizontal velocity	0.825	9.752	<0.0001	2
Neutered-No	0.845	8.411	<0.0001	3
B2-Shoulder-Horizontal velocity	0.864	7.244	<0.0001	4
Proportion HV/BW	0.867	7.080	<0.0001	5
B2-Hind fetlock-Vertical acceleration	0.873	6.672	<0.0001	6
Weight in cannon index	0.882	6.154	0.000	7
B2-Carpus-Total vertical displacement	0.891	5.635	0.000	8
B1-Knee-Total vertical displacement	0.903	4.954	0.001	9
B1-Carpus-Vertical velocity	0.910	4.566	0.002	10
B3-Shoulder-Acceleration	0.911	4.504	0.002	11
B1-Hind fetlock-Total vertical displacement	0.913	4.410	0.002	12
B1-Hip-Horizontal acceleration	0.917	4.172	0.003	13
B3-Knee-Vertical velocity	0.918	4.107	0.003	14
B2-Scapula-Total vertical displacement	0.923	3.855	0.005	15
B2-Elbow-Horizontal velocity	0.924	3.786	0.006	16
Body ratio	0.934	3.255	0.013	17
B2-Tarsus-Horizontal velocity	0.935	3.201	0.014	18
B3-Hip-Vertical velocity	0.937	3.083	0.017	19
Angle 5	0.943	2.771	0.029	20
B1-Iliac crest-Horizontal acceleration	0.943	2.756	0.029	21
Angle 7	0.945	2.692	0.033	22
B2-Scapula-Horizontal velocity	0.946	2.613	0.037	23
B2-Hip-Horizontal velocity	0.947	2.552	0.041	24
B2-Hip-Total vertical displacement	0.950	2.446	0.048	25
B3-Scapula-Horizontal acceleration	0.950	2.437	0.049	26
Angle 4	0.952	2.325	0.058	
B2-Knee-Acceleration	0.953	2.272	0.063	
B1-Hip-Horizontal position	0.954	2.235	0.067	
B2-Fore fetlock-Acceleration	0.954	2.211	0.069	
B2-Hip-Vertical acceleration	0.954	2.198	0.071	
B3-Iliac crest-Vertical velocity	0.957	2.082	0.085	
B2-Iliac crest-Horizontal position	0.958	2.037	0.091	
B1-Hind fetlock-Vertical velocity	0.965	1.683	0.156	
B2-Iliac crest-Total vertical displacement	0.965	1.662	0.161	
B2-Elbow-Acceleration	0.965	1.658	0.162	
B2-Shoulder-Horizontal position	0.970	1.437	0.223	
B1-Elbow-Vertical acceleration	0.970	1.400	0.236	
Chest height index	0.971	1.378	0.243	
B2-Shoulder-Vertical velocity	0.974	1.237	0.297	
B1-Knee-Vertical acceleration	0.981	0.904	0.463	
B2-Scapula-Horizontal position	0.981	0.874	0.480	
B1-Scapula-Vertical acceleration	0.983	0.781	0.539	
B2-Fore fetlock-Vertical acceleration	0.993	0.314	0.869	

Df1 = 4; Df2 = 184.

A Press’ Q value of 17.19 (*n* = 189; *n*’ = 15; *K* = 5) was computed for qualitative gait evaluation levels. Thus, predictions can be considered to be better than chance at 95% (35).

Centroids from different levels for qualitative description of gait performance considered in this study are calculated. The relative position of each centroid is determined by substituting the mean value for the observations depicted in each of the five discriminant functions significantly detected (F1 to F5). The results for the functions at the centroids are reported in Table 4.

**Table 4:** Functions at the centroids for the 13 discriminant functions detected in this study.

Function/Gait qualitative evaluation level	1	2	3	4	5
F1	−476.738	−52.340	40.573	−54.118	134.521
F2	108.758	−29.110	4.553	−11.968	62.230
F3	22.288	10.686	6.627	−17.247	−25.252
F4	1.326	−16.244	3.854	3.913	−23.801
F5	0.000	0.000	0.000	0.000	0.000
F6	0.000	0.000	0.000	0.000	0.000
F7	0.000	0.000	0.000	0.000	0.000
F8	0.000	0.000	0.000	0.000	0.000
F9	0.000	0.000	0.000	0.000	0.000
F10	0.000	0.000	0.000	0.000	0.000
F11	0.000	0.000	0.000	0.000	0.000
F12	0.000	0.000	0.000	0.000	0.000
F13	0.000	0.000	0.000	0.000	0.000

### 3.3 Data mining CHAID decision tree

The CHAID decision tree is presented in Supplementary Figure S1.

## 4 Discussion

Following the premises of Maloiy et al. (24), who stated that locomotory performance is essentially modulated by heritable characters, in lesser proportion by training, for both camels and equines, the present study has investigated which biomechanical and animal morphometric, physiology, and phaneroptic-related variables influence gait performance in dromedary camels leisure riding and racing activities. The mathematical function that had the best fit to a series of data points obtained from the analysis of different gaits (walking, pacing, and galloping) in the studied dromedary camels was identified as a preliminary requirement to effectively study these potential associations. The mathematical model that best fitted (comparatively superior average values of individual *R*^2^; ≥0.7) the dynamics of camel locomotion was the cubic function, whose end behavior at the graphs is determined by the sign of their leading coefficients. These results are in accordance with previous research studies aimed at the mathematical and physical modeling of animal-like locomotion through the use of bioinspired robots (36–39), rigid body models (40), and video analysis (41).

The posterior examination of the statistical relationships between the coefficients of the individual curves of movement and other animal attributes and traits with gait performance made possible the identification of those variables that most largely influence this functional character. On the one hand, the kinematic parameters “vertical velocity”, “total vertical displacement”, “horizontal velocity”, “vertical acceleration”, “acceleration”, “horizontal position”, and “horizontal acceleration” appeared to exert a significant effect on the good performance of camel’s gaits when performing leisure riding and racing activities. In addition to the benefits of good speed and acceleration for covering a specific distance in the shortest time possible (42, 43), the “vertical displacement” and the “horizontal position” of certain body regions during locomotion play a fundamental role in the physical performance of these animals. The vertical displacement determines both the shape and magnitude of the impulse created (vertical jump) and weightlifting performance (44, 45). In regards to the horizontal position, this variable could be an indirect reliable measure of joint rotation and skeletal impairments, thus an indicator of stabilization, alignment, and muscular force of the different body areas implicated in locomotion (46, 47). Therefore, overall athletic performance in leisure riding and racing dromedary camels may be the result of the sum of joint powers and other different sports performance characteristics such as velocity, acceleration, jumping, and change of direction.

Such performance characteristics are known to be intrinsically modulated by linear and angular morphometric measurements, as well as animal physiology, in several vertebrate species (48–57). Specifically, based on our results, superior performance in camel gait is likely to be affected by the angle between the carpus and the fetlock on the forelimb (“angle 4”), the angle between the iliac crest and the greater trochanter of the femur (“angle 5”), the angle between the stifle joint and the tarsus (“angle 7”), the proportion hump volume/body weight (“HV/BW”), the weight in cannon index, the chest height index, the body ratio, and the neutering status of the animals.

Similar to other quadrupedal animals that are bred for athletic activities, such as horses and dogs, the skeletal structure and deviations of the vertical line of carpus/tarsus joint, either medially or laterally, cranially or caudally, would significantly predispose the camel to carpus/tarsus lameness and splints, thus negatively affecting the gait score (58–60). Additionally, several authors confirmed the prominent role of the fore and hind fetlock joint angles, stifle and hock joint angle, and pelvis inclination on weight absorption, energy of propulsion, and storage of elastic strain in riding horses (61–64). Closely related to pelvis inclination, camels are known to be the only animal species that bend the thigh when moving (65). In addition, chest development (“chest height index”) and tallness (“body ratio”) are reported to affect the static/dynamic balance and physical resistance in sport horses (66–69) and athletes (70).

Furthermore, if the hump of a camel is mechanically compared to the size and position of a rider on a horse, it could be inferred that the relative weight and dimensions of the hump (relation HV/BW) have a significant effect on camel locomotion given its influence on body balance, force distribution, and location of the center of gravity, analogous to the effects that the rider-horse-saddle aggregation has on equine gait (71). Moreover, the weight in the cannon index is expected to have a prominent effect on camel locomotion, as the study of the evolutionary history of these animal species has recently revealed. Through the analysis of the vertical ground reaction force with body mass in camelid species, Clemente et al. (72) found that camels have large foot contact areas due to broad fat pads. This condition would decrease the loading rate, thus leading to lower peak foot pressures and musculoskeletal stresses on the limbs. It also helps maintain proportional forces as animals grow in body mass or accelerate. These authors also encountered that dromedary camels load their forelimbs more than their hindlimbs, revealing the higher peak forces in forelimbs and, thus, their major functional relevance in this animal species’ locomotion. Hence, this adaptive trait may be correlated with the afore-discussed prominent role of the alignment of the distal part of the forelimbs and the fore fetlock joint angle on the weight absorption and minimization of mechanic impact during camel locomotion.

In conclusion, the correlation between physiology and function was revealed by the significant impact of the neutering status of the camels on gait performance. In fact, different reports have discussed the advantages of castration in dromedary camels. Some authors highlight the larger effect of castration on the body development in this species if carried out after the animals have reached sexual maturity (73, 74). Concretely, gelded camels are larger, more robust, and more enduring (75, 76). In addition, Iglesias Pastrana et al. (77) remarked an increased effect of neutering on camel organic development and behavior if the animals are castrated not only when they have reached their sexual maturity but have also been initiated in the training protocol for their functionality (e.g., leisure riding). Hence, apart from the benefit of body growth and endurance, castration also has effects on camel behavior, which ultimately constitutes a further criterion of notable interest for functional selection in working animals.

However, other morphometric, phaneroptic, and performance-type variables such as the angle between the cranial side of the scapula and the shoulder joint (“angle 1”), the angle between the shoulder joint and the olecranon (“angle 2”), the angle between the olecranon and the carpus (“angle 3”), the angle between the greater trochanter of the femur and the stifle joint (“angle 6”), the angle between the point of the hock (tarsus) and fetlock (metatarsophalangeal joint) on the hindlimb (“angle 8”), body weight, hump volume, hump weight, density of fat, proportion hump weight/body weight, chest index, cephalic index, pelvic index, dactilothoracic index, conformation index, compactness index, pull force, load force, power, sex, age, coat color and particularities, eye color, owner, training regime, and type of gait did not have a significant discriminatory effect on camel gait performance in leisure riding and racing activities.

First, the aforementioned singular function of fore fetlock joint angles, stifle and hock joint angle, and pelvis inclination on weight absorption, energy of propulsion, and storage of elastic strain in riding animals could be explaining the lack of large impact of the remaining angular measurements evaluated for its potential implication in camel locomotion in the present study. Concerning the effects of body weight, hump volume and weight, and the proportional relationship between different body regions in camel gait performance, the absence of significant affectation of this functional trait by the mentioned factors partially resembles the results of de Oliveira Bussiman et al. (78) for Brazilian horses. However, this contrasts with the results reported by Pratt-Phillips and Munjizun (79) and Gómez et al. (80). These authors support the effects of body mass index, body composition profile, and harmony of physical development on the exercise performance of sport horses, probably due to a combined effect between inflammation-type responses and the impacts of excessive weight carriage on limb health. Then, the contrasting results obtained for camels can be ascribed to different species-specific adaptive traits.

Fatty tissue in camels is mostly concentrated on the hump to improve heat removal through the skin, and the hump size is related to the general reserve status (body condition score) of the animals (81). For this reason, the hump volume in relation to the body weight, and neither the volume and weight of the hump nor the body weight considered separately, significantly influences the locomotion of these animals. Such a condition derives from the implication of the proportionality between the hump volume and the body weight for maintaining the static/dynamic balance of the body of the camel. In addition, in the case of hump weight, this variable greatly depends on the specific fatty acid composition (82), so a heavy hump does not necessarily mean a large volume hump. Apart from that, body balance is strongly controlled by the aforementioned functionality of the broad foot pads, which are responsible for solving the differential impact of the animal’s weight and the speed of movement on the locomotor pressures that affect the musculoskeletal system forces. Finally, in the case of the variables that relate different measurements corresponding to the dimensions of the trunk and extremities, their non-significant influence could also be explained by purely evolutionary reasons. Camels are provided with relatively narrow bodies, long legs, no flank fold that makes the movement of hindlimbs difficult, a less rounded abdomen to allow the hindlimbs freer motion, and limbs more closely to the midline to minimize oscillation between strides, as a result of natural selection for energy efficiency of the movement (9, 83). In close relationship with this last statement, from a pure evolutionary physiology background, pull force, load force, and power may have had a non-significant effect on camel gait performance.

Within the same phylogenetic background, sex, age, coat color and particularities, eye color, owner, training regime, and type of gait did not influence in a significant manner the physical performance of camels. When walking, camels move their ipsilateral limbs in unison (“pacing gait”, as referred to in the literature). Tracing back camel evolutive history, pacing gait is agreed to have been developed about 20 million years ago (84). Apart from the fact that the legs of camels cannot collide at this pacing gait, the stride length, speed, and kinematics efficiency are higher when compared to other gaits, such as regular trots. However, pacing gait made the animal more prone to lateral instability. This way, camels have developed a broad foot pad and a digitigrade stance to abate such relative lack of instability (18, 65). Contrary to horses, camels are known to be the only ungulate species that always use this pattern of leg movements independent of speed (18). Moreover, this is a highly conserved ancestral trait (85). Hence, the underlying genetic basis of camel gaits, which is elementally implicated in the sort of neural impulses from the spine to the extremities (86) and presumably free of epistatic interactions from genes that control coat and eye color associated traits, may be extrapolated to the whole range of physical activities that are feasible for camels to perform. This last hypothesis could be confirmed in future approaches aimed at studying which genomic variants are statistically associated with an enhanced or decreased physical performance.

Then, these statements are contrary to the results presented by Al-Shorepy (14) for racing camels, Entin (87) for racehorses and Greyhound dogs, Vicente et al. (88) for classical riding horses, and Navas González et al. (33) for assisted-therapy donkeys, who reported a significant effect of sex and age on animal functional performance but agreed with Senefeld et al. (89), who support the idea that large differences in athletic performance do not exist between sexes in animals. Regarding the association between coat and eye color with different psychological constructs that are transcendental for animal trainability and safe interaction, the molecular basis of these desirable traits has been identified mainly for dogs and horses (90–93), and some inferences are discussed by Iglesias Pastrana et al. (77) for dromedary camels. Furthermore, a non-negligible impact of rider fitness and interaction with the animal (94), training regime (95, 96), and type of gait (97) on exercise performance are also recognized in the literature on sport horses and dogs. Such controversy could definitively be ascribed to methodological issues (e.g., type and number of potential influencing factors considered, and statistical treatment of data), as well as to animal species and breed-mediated effects.

In summary, rather than the morphology of a specific region of the body, which is correlated with the weight of the animal (98), or factors such as sex, age, and phaneroptic-related variables, known to affect athletic performance in some animal species, a reduced number of angular morphometric measurements, definite body proportions that can affect the general balance of the body, the weight supported by structures with notable involvement in the damping of the mechanical forces produced during locomotion, post-neutering effects, and the particular mobility of specific joints are the factors that should be considered when selecting leisure riding and racing dromedary camels for gait proficiency. More specifically, based on the data mining decision tree, the variables that serve to discriminate with greater accuracy between leisure riding and racing camels for gait performance are the neutering status, with the advantage for neutering, and the kinematic behavior of the scapula, shoulder, carpus, hip, and foot, anatomical structures of singular importance for propulsion, movement direction, and limb support in quadrupeds, as cited above.

With special emphasis on kinematics, the horizontal position of the scapula, the total vertical displacement of the carpus, the horizontal velocity of the shoulder, and the horizontal acceleration of the hip in neutered animals, the horizontal and vertical acceleration of the scapula, and the total vertical displacement of foot in non-neutered animals are specific discriminating parameters among good and poor performance camels. It can also be observed that coefficient 2 is more relevant in castrated animals, while coefficients 1 and 3 are more discriminative among non-castrated animals. Based on the graphic behavior of the cubic function, coefficients 1 and 3 for curves of movement could be related to the direction and force of the movement, while coefficient 2 would be with the amplitude of the movement, suspension duration, and speed (99). From the viewpoint of athletic performance, these results may be indicative of better dressage/beauty and endurance riding performance for intact and castrated animals, respectively. In any case, the selection of the animals should be complemented by the use of behavior evaluation tools. Indeed, the common practice of castration in the camel breed studied in the present research consists of the neutering of the animals once they are initiated in the domestication protocol for leisure riding activities and are sexually mature. It is pursued that animals have reached proper general organic maturity by the mediation of plasma androgens (mainly within brain structures) while also avoiding harmful levels of these hormones from an ethological perspective (77, 100).

With regard to the quantitative evaluation of kinematic parameters, better gait performance in leisure riding and racing camels is achieved when the horizontal position of the scapula is greater than 0, the total vertical displacement of the carpus is greater than −3.78, the horizontal velocity of the shoulder is less than or equal to 2.25, and the horizontal acceleration of the hip is greater than 5.74, for neutered animals. For non-neutered animals, optimal gait performance is observed when the acceleration of the scapula is less than or equal to 0 as well as the total vertical displacement of the foot greater than −2.24. Elementally, the total vertical displacement of the carpus, as an anatomical structure significantly involved in forelimb mechanical support and cushioning, would act as a corrector for the horizontal position of the scapula as well as the horizontal velocity of the shoulder, thus achieving greater general stability, harmony, and energy efficiency of the movement (101). In the case of the horizontal acceleration of the hip, the higher its value, the greater the propulsion force and amplitude of the movement. Contrastingly, for intact camels, a lower acceleration of the scapula and a greater vertical displacement of the distal region of the hindlimbs determine a more harmonious and efficient movement. These values, being interpreted on account of the relative significance of coefficients 1 and 3 for this animal subgroup, reflect the relevance of the rear quarters in maintaining the dynamic balance of the body while limiting as far as possible a wide acceleration at the anterior third, in animals presumably more apt for riding/dressage activities. These functionalities make rear quarters broad, deep, and heavy to support load carriage while moving in an efficient way (102).

Concerning the exploration of new potential functional niches, the results of the present study can assist the tasks of camel valorization and selection for their active participation in animal-assisted therapy since those biomechanical and physiological traits of the animals that most influence locomotor performance, regardless of the type of gait, are identified. On the contrary, for equines, since the cadence of movements can vary between gaits, it may be necessary to look at different and varied selection criteria depending on the type of gait involved in each of the assisted activities in which these animals would be involved. Furthermore, camels are less flighty than horses (103), and the pelvic movement of a person when riding a camel is reported to be very similar to natural human pelvic movements during walking (104); thus, therapeutic applications of camelback riding apart from mere leisure are clearly plausible.

Ultimately, some limitations can be identified in this study. Specifically, future applied studies are encouraged to correlate biomechanical traits with physiological indicators, such as activity-dependent hematological and biochemical parameters, to enhance dromedary camel breeding programs for physically demanding activities not only from a genetic improvement perspective but also to ensure the maintenance of optimal welfare status of the animals.

## 5 Conclusion

Individual curve estimation regression analysis plays a pivotal role in synthesizing complex datasets by constructing validated mathematical descriptions of locomotive behavior in camels. The posterior study of the relationships among morphology, physiology, phaneroptic, and gait performance-related traits made possible the identification of those factors that largely influence gait proficiency in dromedary camels; thus, these criteria should assist breeding decisions for camels dedicated to different athletic activities. Angularity and mechanical forces at distal fore and rear extremity areas, inclination of the pelvis, specific proportionalities affecting the general balance of the body, neutering-mediated effects, and the velocity, acceleration, position, and displacement of particular body regions significantly affect the overall athletic performance in dromedary camels. More specifically, the kinematic behavior of the scapula, shoulder, carpus, hip, and foot, as these anatomical structures primarily control the take-off of the rear limbs and the impact forces of the front limbs, has a prominent impact on the locomotor performance in dromedary camels. Other animal and environment-dependent variables do not influence camel gait performance, probably due to the mixed effect of different species-specific adaptive traits inherited in response to desert environment demands, such as the pacing gait, broad foot pads, and energy efficiency of the movement.

## Data availability statement

The original contributions presented in the study are included in the article/Supplementary material, further inquiries can be directed to the corresponding author.

## Ethics statement

All farms included in the study followed specific codes of good practices and therefore, the animals received humane care in compliance with the national guide for the care and use of laboratory and farm animals in research. Written consent from the owners was obtained for their participation. The study was conducted in accordance with the Declaration of Helsinki. The Spanish Ministry of Economy and Competitivity through the Royal Decree Law 53/2013 and its credited entity, the Ethics Committee of Animal Experimentation from the University of Córdoba, permitted the application of the protocols present in this study as cited in the 5th section of its 2nd article, as the animals assessed were used for credited zootechnical use. This national Decree follows the European Union Directive 2010/63/UE, from the 22 September of 2010.

## Author contributions

CI: Conceptualization, Data curation, Formal analysis, Investigation, Methodology, Software, Validation, Writing – original draft, Writing – review & editing. FN: Conceptualization, Data curation, Formal analysis, Investigation, Methodology, Resources, Software, Supervision, Validation, Visualization, Writing – original draft, Writing – review & editing. EC: Funding acquisition, Investigation, Project administration, Resources, Supervision, Validation, Visualization, Writing – review & editing. CM: Data curation, Formal analysis, Funding acquisition, Resources, Software, Validation, Visualization, Writing – review & editing. JD: Formal analysis, Funding acquisition, Project administration, Resources, Supervision, Validation, Visualization, Writing – review & editing.
