# Supplementary material for: Determination of breeding criteria for gait proficiency in leisure riding and racing dromedary camels: a stepwise multivariate analysis of factors predicting overall biomechanical performance
Source: Front Vet Sci. 2024 Jan 16;10:1297430. doi: 10.3389/fvets.2023.1297430 (PMC10826703; doi:10.3389/fvets.2023.1297430)
Supplement: Supplementary file 1 [file Table_1.DOCX]

**Supplementary Table S1.** Standardized canonical discriminant function coefficients.

| Dependent variable | F1 | F2 | F3 | F4 |
| --- | --- | --- | --- | --- |
| B3-Shoulder-Acceleration | -0.214 | 0.446 | 0.560 | 0.111 |
| B2-Elbow-Acceleration | -0.070 | 0.152 | 0.183 | -0.385 |
| B2-Fore fetlock-Acceleration | 0.257 | 0.121 | -0.044 | 0.169 |
| B2-Knee-Acceleration | -0.188 | -0.395 | -0.437 | -0.237 |
| B3-Scapula-Horizontal acceleration | -0.033 | -0.703 | -0.005 | 0.367 |
| B1-Iliac crest-Horizontal acceleration | 0.510 | -0.155 | 0.172 | -0.161 |
| B1-Hip-Horizontal acceleration | 0.149 | 0.630 | -0.048 | 0.523 |
| B2-Scapula-Horizontal position | 0.097 | 0.044 | -0.266 | 0.185 |
| B2-Shoulder Horizontal position | -0.082 | 0.127 | -0.118 | 0.433 |
| B2-Iliac crest-Horizontal position | -0.341 | 0.243 | 0.106 | 0.136 |
| B1-Hip-Horizontal position | 0.085 | 0.330 | -0.276 | 0.593 |
| B2-Scapula-Horizontal velocity | -0.147 | 0.413 | 0.371 | 0.020 |
| B2-Shoulder-Horizontal velocity | 0.313 | -0.145 | -0.025 | -0.010 |
| B2-Elbow-Horizontal velocity | 0.392 | 0.085 | 0.305 | 0.625 |
| B2-Iliac crest-Horizontal velocity | -0.029 | -0.099 | 0.347 | 0.265 |
| B2-Hip-Horizontal velocity | 0.344 | -0.270 | 0.439 | -0.292 |
| B2-Tarsus-Horizontal velocity | -0.317 | -0.103 | -0.270 | 0.772 |
| B2-Scapula-Total vertical displacement | 0.065 | -0.203 | -0.155 | -0.163 |
| B2-Carpus-Total vertical displacement | 0.819 | 0.640 | -0.230 | -0.246 |
| B2-Iliac crest-Total vertical displacement | -0.338 | 0.447 | 0.244 | -0.141 |
| B2-Hip-Total vertical displacement | -0.463 | -0.009 | -0.261 | 0.009 |
| B1-Knee-Total vertical displacement | -0.637 | -0.066 | 0.049 | -0.401 |
| B1-Hind fetlock-Total vertical displacement | -0.948 | -0.229 | 0.273 | 0.082 |
| B1-Scapula-Vertical acceleration | -0.170 | -0.085 | 0.277 | 0.595 |
| B1-Elbow-Vertical acceleration | -0.080 | 0.243 | 0.310 | -0.696 |
| B2-Fore fetlock-Vertical acceleration | -0.126 | 0.303 | 0.065 | 0.076 |
| B2-Hip-Vertical acceleration | -0.764 | -0.038 | 0.073 | -0.125 |
| B1-Knee-Vertical acceleration | -0.312 | -0.049 | -0.251 | -0.145 |
| B2-Hind fetlock-Vertical acceleration | -0.184 | 0.648 | -0.048 | -0.016 |
| B2-Shoulder-Vertical velocity | -0.235 | -0.483 | -0.409 | 0.013 |
| B1-Elbow-Vertical velocity | -0.687 | -0.071 | 0.095 | 0.184 |
| B1-Carpus-Vertical velocity | -0.626 | -0.548 | 0.475 | -0.118 |
| B3-Iliac crest-Vertical velocity | 0.098 | 0.047 | -0.043 | 0.147 |
| B3-Hip-Vertical velocity | -0.151 | 0.161 | -0.293 | -0.636 |
| B3-Knee-Vertical velocity | 0.469 | 0.263 | 0.194 | 0.075 |
| B1-Hind fetlock-Vertical velocity | -0.234 | -0.014 | 0.044 | 0.194 |
| Angle 4 | -0.086 | 0.238 | -0.233 | -0.004 |
| Angle 5 | 0.023 | 0.276 | -0.212 | 0.465 |
| Angle 7 | -0.182 | 0.064 | 0.393 | -0.145 |
| Proportion HV/BW | -0.397 | -0.199 | 0.063 | -0.155 |
| Weight in cannon index | 0.241 | -0.308 | -0.208 | 0.271 |
| Body ratio | 0.030 | 0.182 | 0.007 | 0.532 |
| Chest height index | -0.162 | 0.009 | -0.126 | -0.337 |
| Neutered-No | -0.362 | 0.374 | -0.597 | 0.044 |
| Neutered-Yes | 0.000 | 0.000 | 0.000 | 0.000 |
