# Supplementary figures and images for: Determination of breeding criteria for gait proficiency in leisure riding and racing dromedary camels: a stepwise multivariate analysis of factors predicting overall biomechanical performance
Source: Front Vet Sci. 2024 Jan 16;10:1297430. doi: 10.3389/fvets.2023.1297430 (PMC10826703; doi:10.3389/fvets.2023.1297430)

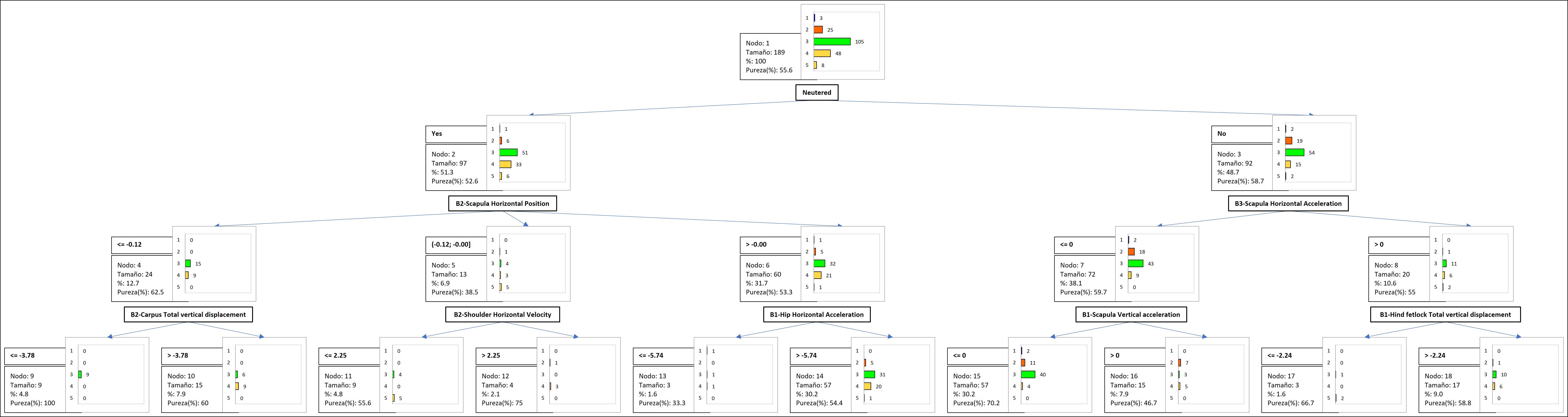

Supplement: Supplementary file 2 [file Image_1.JPEG]
